# Study on Dynamic Behavior of Unmanned Surface Vehicle-Linked Unmanned Underwater Vehicle System for Underwater Exploration

**DOI:** 10.3390/s20051329

**Published:** 2020-02-29

**Authors:** Mai The Vu, Mien Van, Duc Hong Phuc Bui, Quang Thang Do, Tuan-Tu Huynh, Sang-Do Lee, Hyeung-Sik Choi

**Affiliations:** 1 School of Intelligent Mechatronics Engineering, Sejong University, 98 Gunja-dong, Gwangjin-gu, Seoul 143-747, Korea; maithevu90@sejong.ac.kr; 2 School of Electronics, Electrical Engineering and Computer Science, Queen’s University, Belfast BT7 1NN, UK; m.van@qub.ac.uk; 3 Department of Mechanical Engineering, University of Tulsa, Tulsa, OK 74104, USA; hongphucmo@gmail.com; 4 Department of Naval Architecture and Ocean Engineering, Nha Trang University, Nha Trang 650000, Vietnam; thangdq@ntu.edu.vn; 5 Department of Electrical Engineering, Yuan Ze University, No. 135, Yuandong Road, Zhongli 320, Taoyuan 32003, Taiwan; huynhtuantu@saturn.yzu.edu.tw; 6 Department of Electrical Electronic and Mechanical Engineering, Lac Hong University, No. 10, Huynh Van Nghe Road, Bien Hoa, Dong Nai 830000, Vietnam; 7 Department of Ship Operation, Korea Maritime and Ocean University, 727 Taejong-ro, Yeongdo-gu, Busan 49112, Korea; oksangdo@naver.com; 8 Division of Mechanical Engineering, Korea Maritime and Ocean University, 727 Taejong-ro, Yeongdo-gu, Busan 49112, Korea

**Keywords:** unmanned surface vehicle (USV), umbilical cable (UC), unmanned underwater vehicle (UUV), maneuvering

## Abstract

This paper focuses on motion analysis of a coupled unmanned surface vehicle (USV)–umbilical cable (UC)–unmanned underwater vehicle (UUV) system to investigate the interaction behavior between the vehicles and the UC in the ocean environment. For this, a new dynamic modeling method for investigating a multi-body dynamics system of this coupling system is employed. Firstly, the structure and hardware composition of the proposed system are presented. The USV and UUV are modeled as rigid-body vehicles, and the flexible UC is discretized using the catenary equation. In order to solve the nonlinear coupled dynamics of the vehicles and flexible UC, the fourth-order Runge–Kutta numerical method is implemented. In modeling the flexible UC dynamics, the shooting method is applied to solve a two-point boundary value problem of the catenary equation. The interaction between the UC and the USV–UUV system is investigated through numerical simulations in the time domain. Through the computer simulation, the behavior of the coupled USV–UC–UUV system is analyzed for three situations which can occur. In particular, variation of the UC forces and moments at the tow points and the configuration of the UC in the water are investigated.

## 1. Introduction

The guidance and control of marine vessels is an area of focus within the research community. The use of marine vehicles is increasing rapidly within several fields, such as marine biology, seafloor mapping, oceanography, military use, and in the oil and gas industry, and the autonomy of such vehicles is increasing rapidly [1,2,3,4,5]. A basic and highly applicable task for such marine vessels, both surface and underwater, is to follow a general path to perform some mission.

The major mission of an underwater vehicle system is to collect information from the underwater environment and send it back to the control center via sensors, for which reliable data transmission is required. Currently, the reliability of sensors is one of the most important challenges for worldwide research and is a new research trend in many application areas. Castaño et al. [6] mentioned that the reliability of sensors and remote sensing systems is a key enabling step toward the massive utilization of sensor networks in all application fields from manufacturing up to maritime and aeronautic applications. Many methods with different properties and considerations for sensor system reliability such as Bayesian approaches, fuzzy set theory, Dempster–Shafer evidence theory, and gray group decision-making were recently studied to address the reliability of sensors using artificial intelligence. However, with the current technology available, underwater communication is an important challenge in the field. Generally speaking, acoustic wave, blue light, and tether cable are three main kinds of approaches applied for underwater communication. In particular, in order to have a real-time and reliable underwater communication over such a distance, using a tether cable could be a better solution for the real-time surveillance mission of an autonomous underwater vehicle (AUV) [7,8,9]. However, the motion of a long flexible cable in water is very complex, in addition to the non-linear dynamic motion of the unmanned surface vehicle (USV) and unmanned underwater vehicle (UUV), which makes the motion analysis of the couple even more challenging. A marine umbilical cable (UC) exhibits highly nonlinear characteristics especially in the highly dynamic ocean environment, and practical experimentation with the actual system or a representative full-scale apparatus is not practicable; thus, the analysis of marine UC dynamics typically relies on numerical methods. With the different assumptions and considerations, several methods were applied to study the motion of a cable-tethered vehicle system, which include the analytical method [10], experimental method [11], lumped mass method [12], finite difference method [13], and catenary method. Among them, the simplest way of finding a catenary model is to use static catenary equations [8]. Recently, Jung et al. [14] proposed a new hybrid system that combines the USV–UC–UUV to overcome the cumulative navigation error problem of the underwater robot and the battery problem for long-term operation. Dealing with the attached UC–UUV system, Vu et al. [8] attempted theoretically to apply the interaction force of UC to the UUV system. However, this study focused on analyzing only the dynamic behaviors of the UV under the cable effects; thus, it neglected the motions of the USV system.

Some authors conducted research on the dynamic behavior of the combined motion vehicle and cable. Thus far, however, the complex dynamic characteristics of the integrated USV–UC–UUV system are not yet ascertained; therefore, its design scheme and continuous operation performance are yet to be evaluated, as it is costly and extremely difficult to perform in situ ocean tests. Moreover, an underwater vehicle with a cable system is a nonlinear coupled problem that is difficult to handle. It is also a practical problem for naval architects and ocean engineers; therefore, a practical prediction method for analyzing the interaction between the underwater vehicle and the cable system is needed. Thus, this paper presents an approach to multi-body dynamics modeling of a USV–tether UC–UUV system operating at sea. The integrated system is a combination of unmanned surface vehicle, underwater vehicle, and underwater cable to overcome disadvantages such as position accumulation error, the limit of battery capacity, and inability to secure real-time data of the existing unmanned underwater vehicle.

As the nonlinear dynamic equation is difficult and complex to be solved analytically, a model-based motion simulation is implemented by using a numerical method in this paper. Various model-based control methods were studied, such as model predictive control, optimal control, robust control, and the digital twin (DT) approach. Among these methods, the new digital twin (DT) approach emerged as a key concept for modeling, simulation, and optimization of nonlinear systems, in which some real data are also taken into account during the simulation [15].

In this paper, the numerical scheme developed by Vu et al. [8] is extended and then applied to evaluate the interaction of the communication cable on motions of both vehicles USV and UUV. To do so, efficient dynamic models for subsystems were integrated into the total system. The USV is modeled using 3-degree-of-freedom (DOF) rigid body dynamics, while the motion of the UUV is analyzed in 6-DOF. For UC modeling, the catenary equation to conduct motion analysis of the AUV and cable coupling system is applied, and the shooting method is then used to solve the nonlinear finite differential equations in our numerical simulation scheme to obtain more reliable solving results. Finally, the equations of motion considering the motion correlation between each subsystem are described. Several simulations were carried out using the developed equations of motion, and the movement of the USV, as well as that of the UUV, was also observed according to disturbance generated by the UC. The rest of this paper is organized as follows: Section 2 introduces the structure and hardware composition of both USV and UUV systems. Section 3 explores the dynamic model of the coupled USV and UC system. Both kinematics and kinetics are highlighted in this section. Then, Section 4 describes the UC dynamics and how to apply the UC effects to the vehicles. Next, Section 5 provides an overview of the mathematical modeling of the UUV, considering the interaction forces of the UC. Section 6 presents the motion analysis of the integrated USV–UC–UUV system moving in a series of scenarios by numerical simulation. Finally, Section 7 provides the main contributions and conclusions of the work presented throughout this paper.

## 2. The Coupled USV and UUV System

### 2.1. Structure of the USV

The USV was designed to have three propellers for controlling yaw, sway, and surge motion. Two propellers were mounted horizontally in the direction of the track, and one propeller was mounted vertically to perform dynamic positioning functions. The communication between the USV and the operator was made using radio frequency (RF) and long-term evolution (LTE), and the communication between the USV and the UUV was made by serial communication through the UC.

**Figure 1:**
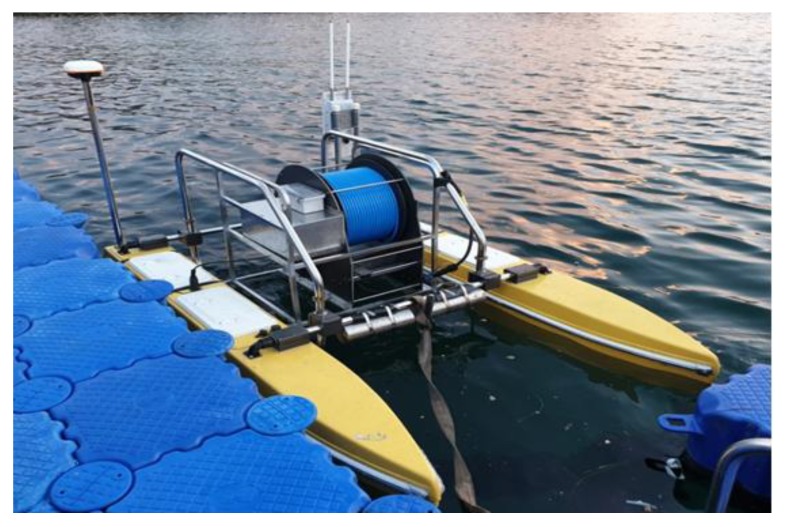
System structure of the unmanned surface vehicle (USV) system.

The structure of the USV is shown in Figure 1. The global positioning system (GPS) was used to measure the position of the USV, and the ultra-short baseline (USBL) was used to find the relative position between the UUV and the USV. The USV consisted of a main control system for the position, attitude, and speed control, a winch system for controlling the depth of UUV, and a communication system for data transmission and reception.

### 2.2. Control System of the USV

**Figure 2:**
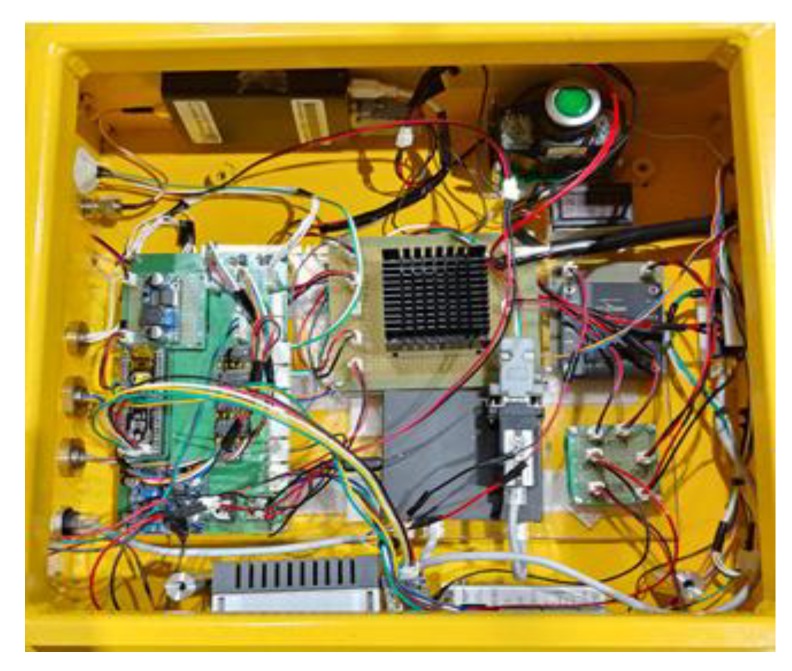
The control box of the USV system.

The control system of the USV consisted of a USV operation control system and a winch control system for controlling depth of the UUV. The system composition is shown in Figure 2.

**Figure 3:**
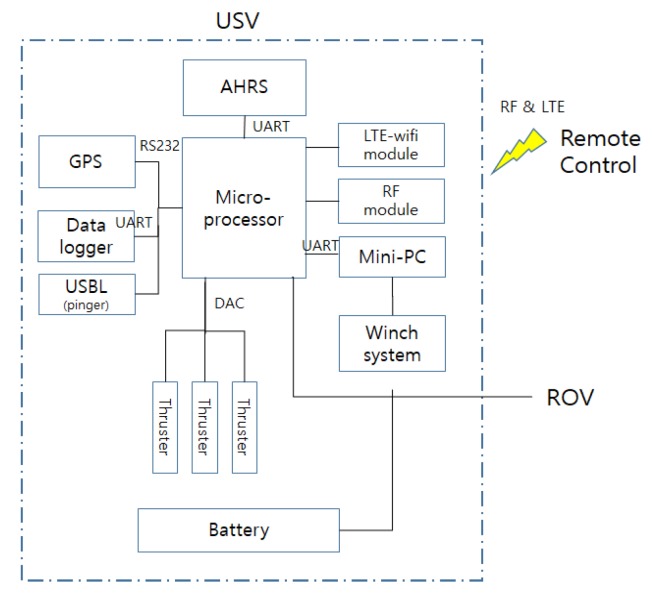
Control system of the USV.

The architecture of the USV control system is shown in Figure 3. An AHRS (attitude heading reference system) and GPS sensor were used to control USV position and attitude, as well as for navigation using the line-of-sight method.

The control signal for the UUV came through the tether cable using serial communication. The winch system was used to control the depth of the UUV. The winch system was installed on the USV as shown in Figure 1.

### 2.3. Structure of the UUV

**Figure 4:**
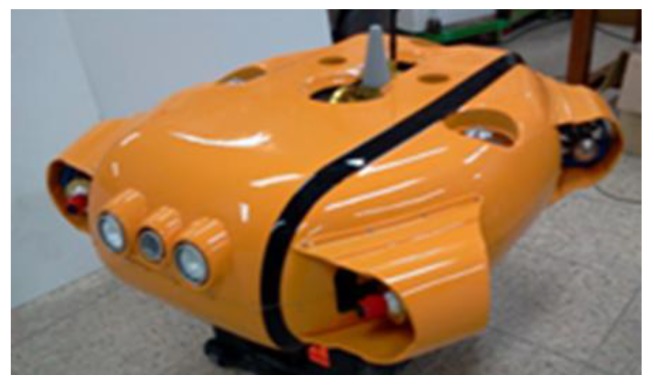
The developed 6-DOF of the unmanned underwater vehicle (UUV).

The shape of the UUV is shown in Figure 4. Three horizontal thrusters were installed at the UUV for surge, sway, and yaw motion control.

**Figure 5:**
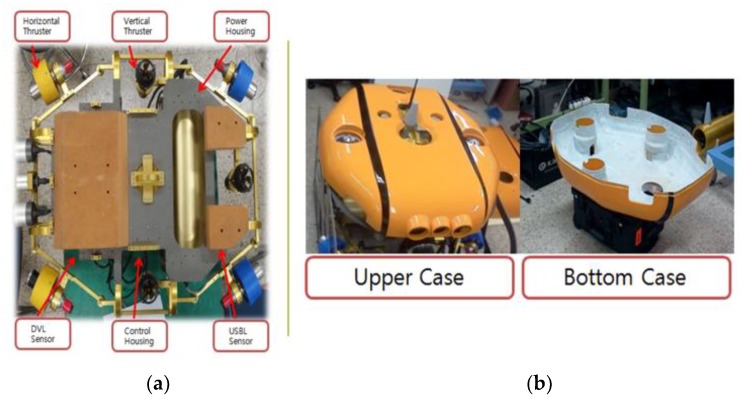
The thruster structure of the UUV: (**a**) internal parts of the UUV; (**b**) Composition of cover.

**Figure 6:**
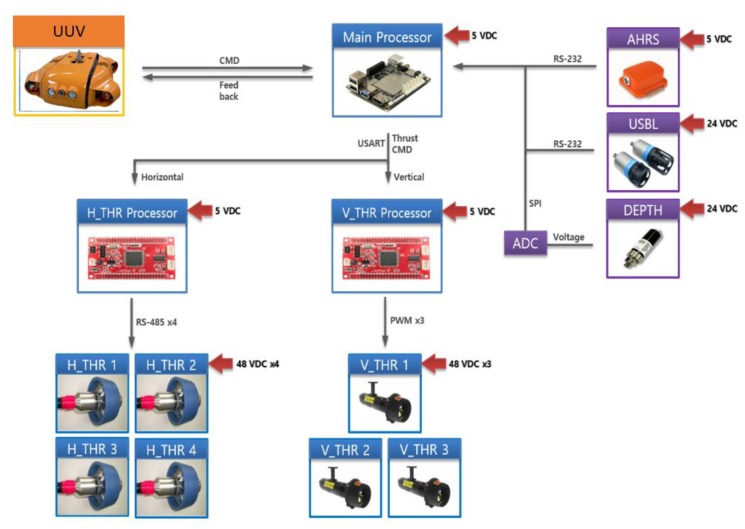
The control structure for the UUV.

The configuration of thrusters in the propulsion system of this UUV is shown in Figure 5. The UUV was designed as an over-actuated system with seven thrusters. The three vertical thrusters were used for heave, pitch, and roll motion, and the four horizontal thrusters were used for surge, sway, and yaw motion. The direction of the thrusters is defined as follows: the four horizontal thrusters were defined as positive where they made a positive contribution in the *x*-direction. Meanwhile, the three vertical thrusters corresponded with a positive *z*-direction contribution. To avoid water being flushed through the UUV, these thrusters were tilted slightly. Figure 6 shows the control structure for the UUV.

## 3. USV Dynamic Modeling

A mathematical model was required for designing the controller of the USV. For this reason, the numerical modeling of the USV is presented in this section. The ocean environment (wind, waves, and currents) which affects the USV dynamics was neglected in this paper.

### 3.1. Assumptions

**Figure 7:**
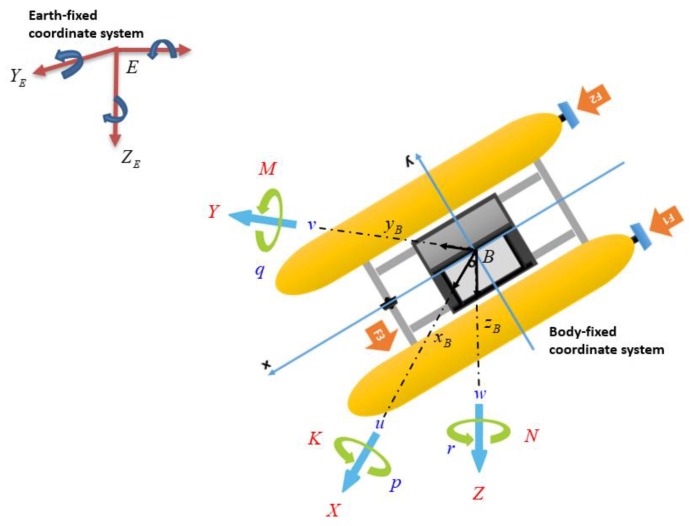
Motion variables for USV.

To simplify the problem, the motion of the USV is described only in the horizontal plane in this paper. The motion variables of the USV in the body-fixed coordinate are shown in Figure 7. Some simplifications were made for computer simulations of the USV motion. These simplifications were as follows:

- The motion of the USV in roll, pitch, and heave directions was neglected.
- The USV had neutral buoyancy and the origin of the body-fixed coordinate was located at the center of mass.
- The USV had three planes of symmetry.
- The dynamic equations of the USV did not include the disturbance forces (waves, wind, and ocean currents).

### 3.2. Three-Coordinate Systems

**Figure 8:**
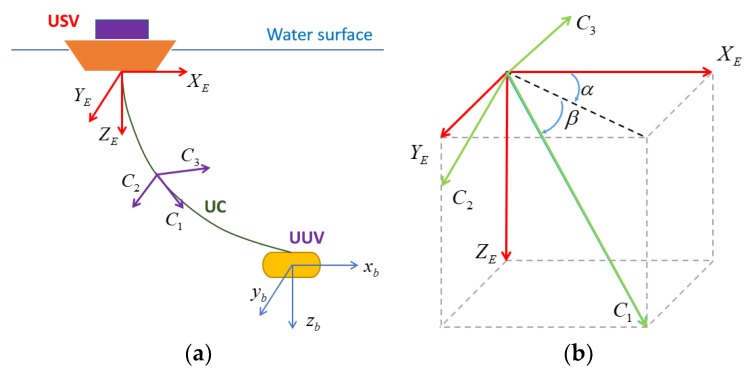
The coordinates of the system: (**a**) coordinates of complete system; (**b**) coordinate of the umbilical cable (UC).

In modeling the complete USV–UC–UUV system in this study, we defined three coordinate systems composed of the earth-fixed coordinate (*X_E_*, *Y_E_*, *Z_E_*), the local cable coordinates along the UC (*C*_1_, *C*_2_, *C*_3_), and the vehicle-fixed coordinate (*x_b_*, *y_b_*, *z_b_*), as shown in Figure 8. Firstly, the earth-fixed coordinate was located at the mass center of the USV, which was defined with *X_E_* pointing to the northerly direction, *Y_E_* pointing to the easterly direction, and *Z_E_* pointing to the earth. Next, the UC was divided into small rigid segments instead of a continuous non-rigid system. Each segment had a coordinate system (*C*_1_, *C*_2_, *C*_3_) with *C*_1_ tangent to the UC, and *C*_3_ in the plane of {*X_E_*, *Y_E_*}, whereas *C*_1_ and *C*_2_ were orthogonal vectors applied to the whole length of the UC. Lastly, the vehicle-fixed coordinate (*x_b_*, *y_b_*, *z_b_*) was defined with respect to the UUV itself. The UUV reference coordinates are described in Figure 8.

With the defined coordinates, the rotation matrix for converting from the UUV coordinate (*x_b_*, *y_b_*, *z_b_*) to the earth-fixed coordinate (*X_E_*, *Y_E_*, *Z_E_*), with regard to Euler angles in Reference [16], can be expressed as follows:

(1) $$\left[\begin{array}{ccc} x_{b} & y_{b} & z_{b} \end{array}\right]=\left[\begin{array}{ccc} X_{E} & Y_{E} & Z_{E} \end{array}\right]R(\phi ,\theta ,\psi ),$$

(2) $$R(\phi ,\theta ,\psi )=\left[\begin{array}{ccc} c\theta c\psi & s\phi s\theta c\psi -c\phi s\psi & c\phi s\theta c\psi +s\phi s\psi \\ c\theta s\psi & s\phi s\theta s\psi +c\phi c\psi & c\phi s\theta s\psi -s\phi c\psi \\ -s\theta & s\phi c\theta & c\phi c\theta \end{array}\right],$$

where ϕ, *θ*, and *ψ* are the roll, pitch, and heading angles of the vehicles, respectively.

Moreover, the transformation from the UC coordinate (*C*_1_, *C*_2_, *C*_3_) to the earth-fixed coordinate (*X_E_*, *Y_E_*, *Z_E_*) can be represented in a simple matrix form as follows [13]:

(3) $$\left[\begin{array}{ccc} C_{1} & C_{2} & C_{3} \end{array}\right]=\left[\begin{array}{ccc} X_{E} & Y_{E} & Z_{E} \end{array}\right]\left[\begin{array}{ccc} c\;\alpha \;c\;\beta & -c\;\alpha \;s\;\beta & s\;\alpha \\ -s\;\alpha \;c\;\beta & s\;\alpha \;s\;\beta & c\;\alpha \\ -s\;\beta & -c\;\beta & 0 \end{array}\right].$$

Considering Equations (1)–(3), the transformation from the UC coordinate (*C*_1_, *C*_2_, *C*_3_) to the UUV coordinate (*x_b_*, *y_b_*, *z_b_*) can be determined as follows:

(4) $$\left[\begin{array}{ccc} C_{1} & C_{2} & C_{3} \end{array}\right]=\left[\begin{array}{ccc} x_{b} & y_{b} & z_{b} \end{array}\right]R^{T}(\phi ,\theta ,\psi )\left[\begin{array}{ccc} c\;\alpha \;c\;\beta & -c\;\alpha \;s\;\beta & s\;\alpha \\ -s\;\alpha \;c\;\beta & s\;\alpha \;s\;\beta & c\;\alpha \\ -s\;\beta & -c\;\beta & 0 \end{array}\right].$$

Note that the rotation matrix, R, is orthogonal and, hence, its inverse is equal to the transpose of its matrix, which is useful for easily converting from earth-fixed coordinates to body-fixed coordinates.

### 3.3. Mathematical Model

A model for a system is not only useful for formulating control algorithms, but also for performing the simulation. According to Reference [9], when deriving a control design model of a USV, it can be assumed that only the motions in the horizontal plane, which are surge motion, sway motion, and yaw motion, are of interest to reduce the model complexities.

To express the kinematics of a USV, two coordinates were defined as shown in Figure 7. The first coordinate $EX_{E}Y_{E}Z_{E}$ describes the earth-fixed coordinate while the second coordinate $Bx_{B}y_{B}z_{B}$ represents the body-fixed coordinate.

Furthermore, using the notation of Society of Naval Architects and Marine Engineers (SNAME) (1950) [17], a USV operating in 3-DOF can be described by six motion variables. The first mode is the position of the USV $\eta ={\left[\begin{array}{ccc} x & y & \psi \end{array}\right]}^{T}\in {ℜ}^{3}$, which is referred to as surge, sway, and yaw motions, and it describes the position of the USV in the horizontal plane with reference to earth-fixed coordinate $EX_{E}Y_{E}Z_{E}$. The second mode is the velocity of the USV $v={\left[\begin{array}{ccc} u & v & r \end{array}\right]}^{T}\in {ℜ}^{3}$, where *u* and *v* are the surge and sway velocities and *r* is the heave velocity with reference to body-fixed coordinate $Bx_{B}y_{B}z_{B}$.

The relationship between position and orientation of the USV in the earth-fixed coordinate ($EX_{E}Y_{E}Z_{E}$) and the linear and angular velocities in the vehicle coordinate ($Bx_{B}y_{B}z_{B}$) is given as

(5) $$\dot{\eta }=R\left(\eta \right)v,$$

where the rotation matrix R(η) is defined as

(6) $$R(\eta )=\left[\begin{array}{ccc} \cos\psi & -\sin\psi & 0\\ \sin\psi & \cos\psi & 0\\ 0 & 0 & 1 \end{array}\right],R^{-1}(\eta )=R^{T}(\eta ).$$

The dynamic model of the USV is based on the model by Fossen [18]. This model of the USV in 3-DOF is derived from the Newton–Euler motion equation as described in Reference [9].

(7) $$M\dot{v}+C(v)v+D(v)v+g(\eta )=\tau +\tau_{cable},$$

where $M\in {ℜ}^{3\times 3}$ is the symmetric positive definite inertia matrix, $C(v)\in {ℜ}^{3\times 3}$ is the centripetal and Coriolis matrix, and $D(v)\in {ℜ}^{3\times 3}$ is the damping matrix; $g(\eta )\in {ℜ}^{3}$ represents the gravitational forces, and we assume that $g(\eta )={\left[0,\;0,\;0\right]}^{T}$, $\tau =\left[\tau_{X},\;\tau_{Y},\;\tau_{N}\right]\in {ℜ}^{3}$ represents the control input, and $\tau_{cable}=\left[\tau_{Cx},\;\tau_{Cy},\;\tau_{Cn}\right]\in {ℜ}^{3}$ represents the cable forces and moments.

Additionally, the matrices *M*, C(v), and D(v) are expressed as

(8) $$M=\left[\begin{array}{ccc} m-X_{\dot{u}} & 0 & 0\\ 0 & m-Y_{\dot{v}} & mx_{g}-Y_{\dot{r}}\\ 0 & mx_{g}-N_{\dot{v}} & I_{z}-N_{\dot{r}} \end{array}\right],$$

(9) $$C(v)=\left[\begin{array}{ccc} 0 & 0 & (Y_{\dot{v}}-m)v+\left(\frac{1}{2}Y_{\dot{r}}+\frac{1}{2}N_{\dot{v}}-mx_{g}\right)r\\ 0 & 0 & \left(m-X_{\dot{u}}\right)u\\ -(Y_{\dot{v}}-m)v-\left(\frac{1}{2}Y_{\dot{r}}+\frac{1}{2}N_{\dot{v}}-mx_{g}\right)r & -\left(m-X_{\dot{u}}\right)u & 0 \end{array}\right],$$

(10) $$D(v)=\left[\begin{array}{ccc} -X_{u}-X_{\left|u\right|u}\left|u\right| & 0 & 0\\ 0 & -Y_{v}-Y_{\left|v\right|v}\left|v\right|-Y_{\left|r\right|v}\left|r\right| & -Y_{r}-Y_{\left|v\right|r}\left|v\right|-Y_{\left|r\right|r}\left|r\right|\\ 0 & -N_{v}-N_{\left|v\right|v}\left|v\right|-N_{\left|r\right|v}\left|r\right| & -N_{r}-N_{\left|v\right|v}\left|v\right|-N_{\left|r\right|v}\left|r\right| \end{array}\right],$$

where the term m describes the dry mass of the USV, *x_g_* is the coordinate between the center of gravity and vehicle origin in the *x*-axis expressed in the body-fixed frame, and the term $I_{z}$ denotes the moments of inertia about the $Bz_{B}$ axis. $X_{\dot{u}}$, $Y_{\dot{v}}$, $Y_{\dot{r}}$, $N_{\dot{v}}$, $N_{\dot{r}}$, $X_{u}$, $Y_{v}$, $Y_{r}$, $N_{v}$, $N_{r}$, $X_{\left|u\right|u}$, $Y_{\left|v\right|v}$, $Y_{\left|r\right|v}$, and $Y_{\left|v\right|r}$, $Y_{\left|r\right|r}$, $N_{\left|v\right|v}$, $N_{\left|r\right|v}$ are the hydrodynamic coefficients of the USV.

### 3.4. Configuration of Thrusters

**Figure 9:**
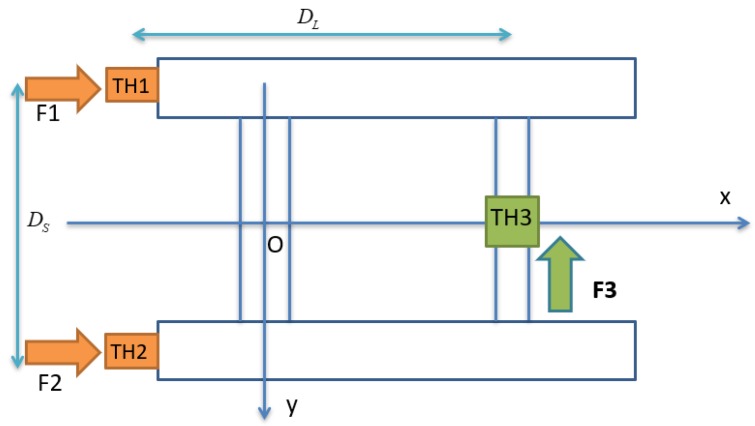
Thruster arrangement of the USV.

The USV was designed to have three thrusters for 3-DOF motion control as shown in Figure 9.

With revolution speed *n_i_*, the thrust force is expressed as shown in Equation (11).

(11) $$T_{i}=\rho n_{i}^{2}D_{P}^{4}K_{T},$$

where ρ is the density of sea water, *D_P_* is the diameter of three thrusters located on the USV, and *K_T_* is the thrust coefficient.

The relationship between local thrust force and body-fixed thrust force on the USV can be described as

(12) $$\tau_{c}=T\cdot F_{T},$$

where the individual thrust force vector is $F_{T}={\left[\begin{array}{ccc} F_{1} & F_{2} & F_{3} \end{array}\right]}^{T}\in {ℜ}^{3\times 1}$, and the generalized force vector acting on the USV is $\tau_{c}={\left[\begin{array}{ccc} F_{x} & F_{y} & M_{z} \end{array}\right]}^{T}\in {ℜ}^{3\times 1}$.

Moreover, the thrust allocation of the USV is expressed as

(13) $$T=\left[\begin{array}{ccc} 1-t_{p} & 1-t_{p} & 0\\ 0 & 0 & 1-t_{p}\\ (1-t_{p})\frac{D_{L}}{2} & -(1-t_{p})\frac{D_{L}}{2} & (1-t_{p})\frac{D_{s}}{2} \end{array}\right],$$

where *t_p_* is the thrust deduction coefficient of each thruster, *D_L_* is the distance between the forward thruster (TH3) and two stern thrusters (TH1, TH2), and *D_s_* is the distance between the two-port thruster (TH1) and starboard thruster (TH2).

## 4. Cable Dynamic Modeling

The UC was used to connect the USV with the UUV and to supply power and communication. However, water resistance to the UC interferes with or restrains the movement of the USV and UUV. In this section, the interacting forces between the USV and UUV are analyzed. For this, the mathematical modeling of the UC motion in water is presented. For modeling of the UC motion in water, the catenary equations and shooting method are proposed.

### 4.1. Assumptions

In this paper, to analyze interaction forces of the UC between the USV and UUV, the following assumptions are proposed:

- A continuous, inextensible, and flexible UC was used in this study.
- The UC had no bending and torsional stiffness.
- The length of the UC was constant L = 100 m.
- The UC acted as the axial force, UC self-weight, and hydrodynamic drag forces.
- The stress/strain of the UC was linear.

### 4.2. Mathematical Model

**Figure 10:**
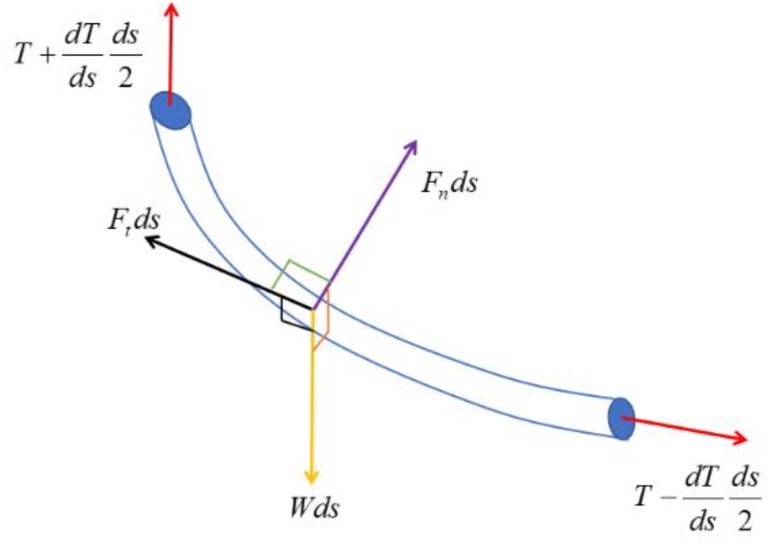
Forces acting on a segment of the UC.

In this paper, when the UC was suspended between the USV and UUV, there were four kinds of forces acting on the UC including gravity forces, buoyancy forces, drag forces, and residual bottom tension, which are described in Figure 10.

External forces on the UC are caused by the environmental forces such as hydrodynamic drag and gravity. In this paper, it is assumed that the attached UC is a long slender pipe, and the drag force acts on the UC. Morison’s equation is used to estimate the forces acting on the UC. Thus, the tangential and normal components of the drag force can be respectively expressed as follows:

(14) $$F_{t}=\frac{1}{2}\rho C_{t}d\left|V_{t}\right|V_{t},$$

(15) $$F_{n}=\frac{1}{2}\rho C_{n}d\left|V_{n}\right|V_{n},$$

where ρ is the density of seawater, and d is the diameter of the cable; *V_t_* and *V_n_* are respectively the tangential and normal components of current velocity relative to the UC. *C_t_* and *C_n_* are respectively the tangential and normal drag coefficients.

The UC has weight such that it satisfies the catenary equation. The static calculation of the shape and tension of an ideal cable was given by Reference [19].

For modeling the UC, the cable with two boundary conditions was divided into many segments along its length. Then, we defined *s_i_* to be the value of *s* in node *I*; thus, the segment *i* was the part of the cable where $s_{i-1}<s\leq s_{i}$. The reaction force *f*_0_ in *s = 0* is described as

(16) $$f_{0}=\sum_{i=1}^{m}w_{i}\left(s_{i}-s_{i-1}\right)+\sum_{i=1}^{m}f_{i},$$

where *m* is the number of segments of the cable, $w_{i}^{i}\in R^{3}$ is the constant distributed force acting over segment *i*, and $f_{i}^{i}\in R^{3}$ is nodal force acting on the nodal point *i*.

The strain of each cable segment ε can be expressed as the function of an infinitesimal cable segment *ds* and the stretched length *dp*.

(17) $$\varepsilon =\frac{dp-ds}{ds}.$$

Moreover, the axial strain can be described by the relationship between the axial tension T, Young’s modulus of cable *E*, and the cross-sectional area of the cable *A*.

(18) $$\varepsilon =\frac{T}{EA}.$$

The cable tension *T(s):* $\left[0,L\right]\to R^{3}$ is estimated from the expression

(19) $$T(s)=T(s){\frac{dr}{dp}|}_{s}=f_{0}-\sum_{i=1}^{m-1}f_{i}-\sum_{i=1}^{m-1}f_{i}\left[w_{i}\left(s_{i}-s_{i-1}\right)\right]-w_{m}\left(s-s_{m-1}\right),$$

where *m* is the number of cable segments divided along the length of the UC, and *dp* is obtained from Equation (17). The position of the cable expressed in coordinate *i* is defined as $r^{i}(s):\left[0,L\right]\to R^{3}$. For simplification, we express

(20) $$F_{m-1}=f_{0}-\sum_{i=1}^{m-1}f_{i},\;\;\;\;\;\;1\leq m\leq N,$$

(21) $$W_{m-1}=\sum_{i=1}^{m-1}w_{i}\left(s_{i}-s_{i-1}\right).$$

The cable tension can be solved from

(22) $$T^{T}(s)T(s)=T^{2}(s)\frac{df^{T}}{dp}\frac{df}{dp},$$

(23) $$T(s)=\sqrt{{\left(F_{m-1}-W_{m-1}-w_{m}\left(s-s_{m-1}\right)\right)}^{T}\left(F_{m-1}-W_{m-1}-w_{m}\left(s-s_{m-1}\right)\right)}.$$

Using Equations (17) and (18) along with the assumption of the cable which has a linear stress–strain relationship, the cable tension can be defined as

(24) $$T(s)=E(s)A(s)\left(\frac{dp}{ds}-1\right).$$

Furthermore, the relationship between the direction of an unstretched length of the cable segment *ds* and a stretched cable segment *dp* can be obtained as

(25) $$\frac{df}{ds}=\frac{df}{dp}\frac{dp}{ds}.$$

By using Equations (23) and (24), Equation (25) can be rearranged as

(26) $$\frac{df}{ds}=\left(F_{m-1}-W_{m-1}-w_{m}\left(s-s_{m-1}\right)\right)\left(\frac{1}{E(s)A(s)}+\frac{1}{\sqrt{{\left(F_{m-1}-W_{m-1}-w_{m}\left(s-s_{m-1}\right)\right)}^{T}\left(F_{m-1}-W_{m-1}-w_{m}\left(s-s_{m-1}\right)\right)}}\right).$$

In order to solve Equation (26), the ordinary numerical integration method can be applied. Sagatun [19] presented the closed-form solution of this integral, which is

(27) $$f_{m}(s)=\frac{K_{1}(s)}{K_{2}{}^{3}}(F_{m-1}K_{2}{}^{2}-F_{m-1}⊗w⊗w-F_{m-1}⊗(P(F_{m-1}⊗w)))-w\frac{1}{K_{2}{}^{2}}T(s)+\frac{1}{EA}\left(F_{m-1}s-\frac{1}{2}ws^{2}\right)+C_{m-1},$$

where the sign ⊗ describes component-wise multiplication. The tension of each cable segment *T(s)* is obtained from Equation (23), where

(28) $$K_{1}(s)=\ln\left(\left(K_{2}s-\frac{1}{K_{2}}F_{m-1}^{T}w\right)+T(s)\right),K_{2}=\sqrt{w^{T}w}={\left\|w\right\|}_{2},P=\left[\begin{array}{ccc} 0 & 1 & 1\\ 1 & 0 & 1\\ 1 & 1 & 0 \end{array}\right].$$

Using the assumption that cable segment 1 is at the point 0, this means that *r(0) = r0*, and the continuities between the segments have to be fulfilled. Then, we have

(29) $$\begin{array}{l} f(0)=0,\\ f{\left(s_{i}\right)}^{-}=f{\left(s_{i}\right)}^{+},\left\{i\in \left[1,n-1\right]\to m\right\} \end{array}$$

The integration constants can be obtained as

(30) $$C_{m-1}=\{\begin{array}{ccc} -f_{0}, & for & m=1\\ f_{m-1}(s_{m-1})-f_{m}(s_{m}), & for & \begin{array}{cc} k\in \left[2,n\right]\to m, & \begin{array}{cc} for & n\geq 2 \end{array} \end{array} \end{array}.$$

### 4.3. Boundary Conditions

In order to solve the governing equation for the UC, two boundary conditions at both ends of the UC were applied. The response of the UC ends was given by the connections to the USV and UUV. In this case, the boundary conditions were placed at both ends of the UC (the upper end attached to the USV and the lower end connected to the UUV). Due to this, the USV motion was on the water surface; thus, the first boundary condition *P_USV_* = *P_USV_ (t)* as a function of time represents the position of the USV during the motion. Similarly, the second boundary condition at the point connected to the UUV *P_UUV_* = *P_UUV_ (t)* describes the position of the UUV, also considered a function of time. Thus, the top and bottom boundary conditions were

(31) $$x_{usv}(0,t)=x_{usv},\;y_{usv}(0,t)=y_{usv},\;z_{usv}(0,t)=z_{usv},$$

(32) $$x_{uuv}(L,t)=x_{uuv},\;y_{uuv}(L,t)=y_{uuv},\;z_{uuv}(L,t)=z_{uuv},$$

where *x_usv_*, *y_usv_*, *z_usv_* and *x_uuv_*, *y_uuv_*, *z_uuv_* represent the positions of the USV and the UUV during their motion, respectively.

### 4.4. Cable Effects

Note that the tension of the UC, T, caused the forces and moments acting on both vehicles (USV and UUV) to vary with time because the positions of vehicles changed with time when the UC moved in the water. The equations below which were mentioned by Reference [8], were used to represent the UC effects on both vehicle dynamics, where the UC forces and moments are expressed in the vehicle-fixed frame.

(33) $$F_{c}(t)=\left[\begin{array}{c} F_{cX}\\ F_{cY}\\ F_{cZ} \end{array}\right]=-R^{T}(\phi ,\theta ,\psi )\left[\begin{array}{ccc} c\;\alpha \;c\;\beta & -c\;\alpha \;s\;\beta & s\;\alpha \\ -s\;\alpha \;c\;\beta & s\;\alpha \;s\;\beta & c\;\alpha \\ -s\;\beta & -c\;\beta & 0 \end{array}\right]\left[\begin{array}{c} T\\ 0\\ 0 \end{array}\right],$$

(34) $$M_{c}(t)=\left[\begin{array}{c} M_{cX}\\ M_{cY}\\ M_{cZ} \end{array}\right]=r_{c}\times F_{c}(t)=\left[\begin{array}{c} x_{c}\\ y_{c}\\ z_{c} \end{array}\right]\times \left[\begin{array}{c} F_{cX}\\ F_{cY}\\ F_{cZ} \end{array}\right]=\left[\begin{array}{c} y_{c}F_{cZ}-z_{c}F_{cY}\\ z_{c}F_{cX}-x_{c}F_{cZ}\\ x_{c}F_{cY}-y_{c}F_{cX} \end{array}\right],$$

where $\vec{r_{c}}=\left(x_{c},y_{c},z_{c}\right)$ denotes the location of the tow points at the vehicles (USV and UUV), expressed in the vehicle frames.

## 5. UUV Dynamic Modeling

A detailed dynamic model of the UUV was used to estimate the vehicle behavior in various situations. In this section, the 6-DOF equation of the motions of the UUV with seven thrusters are described based on the physics of an over-actuated UUV and its actuators.

### 5.1. Assumptions

The dynamic model of the UUV was quite complex and needed many parameters; therefore, the following assumptions were used to simplify the model.

- The UUV was fairly symmetrical about its three planes.
- The center of buoyancy of the UUV was located on the geometric symmetry plane.
- There were no environmental disturbances acting on the UUV.
- The UUV was considered as a rigid body; thus, there were no bending and geometrical deformations.
- The hydrodynamic coefficients of the UUV were not variable.

### 5.2. Mathematical Model

**Figure 11:**
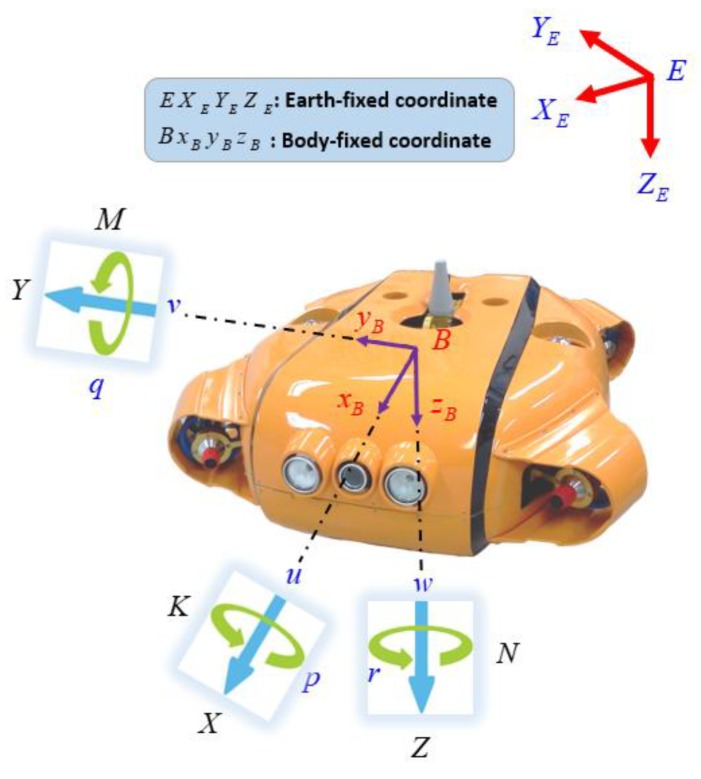
Coordinate system of the UUV.

Following standard practice [18], a 6-DOF nonlinear model of a UUV was expressed using the earth-fixed coordinate and a body-fixed coordinate as shown in Figure 11. The body-fixed coordinate O-*xyz* was attached to the center of the gravity of the vehicle. The motion of the body-fixed coordinate is described relative to the earth-fixed coordinate E-*XYZ*.

The notation used throughout this paper is based on the 6-DOF representation for the UUV, given by Reference [17].

(35) $$\begin{array}{l} \eta ={\left[\begin{array}{cc} \eta_{1}^{T} & \eta_{2}^{T} \end{array}\right]}^{T},\eta_{1}={\left[x,y,z\right]}^{T},\eta_{2}={\left[\phi ,\theta ,\psi \right]}^{T}\\ \nu ={\left[\begin{array}{cc} \nu_{1}^{T} & \nu_{2}^{T} \end{array}\right]}^{T},\nu_{1}={\left[u,v,w\right]}^{T},v_{2}={\left[p,q,r\right]}^{T}\\ \tau ={\left[\begin{array}{cc} \tau_{1}^{T} & \tau_{2}^{T} \end{array}\right]}^{T},\tau_{1}={\left[X,Y,Z\right]}^{T},\tau_{2}={\left[K,M,N\right]}^{T} \end{array}$$

where $\eta_{1}\in {ℜ}^{3\times 1}$ is the linear position of the UUV, and $\eta_{2}\in {ℜ}^{3\times 1}$ is the vector of Euler angles. Both $\eta_{1}$ and $\eta_{2}$ are defined in the earth-fixed coordinate E-*XYZ*. Meanwhile, $v_{1}\in {ℜ}^{3\times 1}$ denotes the translational velocities in surge, sway, and heave motions of the UUV, and $v_{1}\in {ℜ}^{3\times 1}$ denotes the rotational velocities in roll, pitch, and yaw motions in the body-fixed coordinate O-*xyz*. Finally, the vector $\tau \in {ℜ}^{3\times 1}$ describes the generalized forces and moments acting on the UUV in the body-fixed coordinate O-*xyz*, with $\tau_{1}\in {ℜ}^{3\times 1}$ corresponding to the forces along *x-*, *y-*, and *z*-axes, while $\tau_{2}\in {ℜ}^{3\times 1}$ corresponds to the moments about the *x-*, *y*-, and *z*-axes.

The transformation matrix J relates the motion of the UUV in the body-fixed coordinate to the earth-fixed coordinate. Thus, the kinematic equation for the UUV expressed using generalized coordinates in 6-DOF is given as

(36) $$\dot{\eta }=J(\eta_{2})\nu .$$

The rotation matrix between the body-fixed coordinate and the earth-fixed coordinate related to Euler angles is given by

(37) $$J(\eta_{2})=\left[\begin{array}{cc} J_{1} & 0^{3\times 3}\\ 0^{3\times 3} & J_{2} \end{array}\right].$$

The linear velocity transformation matrix $J_{1}$, and the angular velocity transformation matrix $J_{2}$ in Equation (37), related to Euler angles, are obtained by

(38) $$J_{1}\left(\eta_{2}\right)=\left[\begin{array}{ccc} c\psi \;c\theta & -s\psi \;c\phi +s\phi \;s\theta \;c\psi & s\psi \;s\phi +s\theta \;c\psi \;c\phi \\ s\psi \;c\theta & c\psi \;c\phi +s\phi \;s\theta \;s\psi & -c\;\psi s\phi +s\theta \;s\psi \;c\phi \\ -s\;\theta & s\phi \;c\theta & c\phi \;c\theta \end{array}\right],$$

(39) $$J_{2}\left(\eta_{2}\right)=\left[\begin{array}{ccc} 1 & \sin\phi \tan\theta & \cos\phi \tan\theta \\ 0 & \cos\phi & -\sin\phi \\ 0 & \sin\phi /\cos\theta & \cos\phi /\cos\theta \end{array}\right],$$

where the notations s(.) = sin (.), c(.) = cos (.), and t(.) = tan (.) are used for notational brevity. Notice that the transformation matrix of the angular velocity $J_{1}\left(\eta_{2}\right)$ is globally invertible since $J_{1}{}^{-1}\left(\eta_{2}\right)=J_{1}{}^{T}\left(\eta_{2}\right)$.

The nonlinear dynamic equation of the UUV can be presented as a compact matrix form [16].

(40) $$M\dot{v}+C(v)v+D(v)v+G(\eta )=\tau_{th}+\tau_{cable},$$

where $M\in {ℜ}^{6\times 6}$ is an inertial matrix of UUV.$C(v)\in {ℜ}^{6\times 6}$ is a centripetal force and Coriolis matrix. $D(v)\in {ℜ}^{6\times 6}$ is a hydrodynamic damping matrix. $G(\eta )\in {ℜ}^{6\times 1}$ is a gravity and buoyancy term, $\tau_{th}\in {ℜ}^{6\times 1}$ represents the propulsion forces and moments acting on the UUV, and $\tau_{cable}\in {ℜ}^{6\times 1}$ denotes the UC forces and moments. Moreover, the aforementioned matrices are described as follows:

(41) $$M\;=\left[\begin{array}{cccccc} m+X_{\dot{u}} & 0 & 0 & 0 & mz_{G} & -my_{G}\\ 0 & m+Y_{\dot{v}} & 0 & -mz_{G} & 0 & mx_{G}+Y_{\dot{r}}\\ 0 & 0 & m+Z_{\dot{w}} & my_{G} & -mx_{G}+Z_{\dot{q}} & 0\\ 0 & -mz_{G} & my_{G} & I_{xx}+K_{\dot{p}} & I_{xy} & I_{xz}\\ mz_{G} & 0 & -mx_{G}+M_{\dot{w}} & I_{yx} & I_{yy}+M_{\dot{q}} & I_{yz}\\ -my_{G} & mx_{G}+N_{\dot{v}} & 0 & I_{zx} & I_{zy} & I_{zz}+N_{\dot{r}} \end{array}\right],$$

(42) $$\begin{array}{l} C(v)=[\begin{array}{ccc} 0 & 0 & 0\\ 0 & 0 & 0\\ 0 & 0 & 0\\ -m\left(y_{G}q+z_{G}r\right) & m\left(y_{G}q+w\right)+Z_{\dot{w}}w & m\left(z_{G}p-v\right)-Y_{\dot{v}}v\\ m\left(x_{G}q-w\right)-Z_{\dot{w}}w & -m\left(z_{G}r+x_{G}p\right) & m\left(z_{G}q+u\right)+X_{\dot{u}}u\\ m\left(x_{G}r+v\right)+Y_{\dot{v}}v & m\left(y_{G}r-u\right)-X_{\dot{u}}u & -m\left(x_{G}p+y_{G}q\right) \end{array}\\ \;\;\;\;\;\;\;\;\;\;\;\;\;\;\;\;\;\;\;\;\;\;\;\;\;\;\;\;\;\begin{array}{ccc} m\left(y_{G}q+z_{G}r\right) & -m\left(x_{G}q-w\right)+Z_{\dot{w}}w & -m\left(x_{G}r+v\right)-Y_{\dot{v}}v\\ -m\left(y_{G}q+w\right)-Z_{\dot{w}}w & m\left(z_{G}r+x_{G}p\right) & -m\left(y_{G}r-u\right)+X_{\dot{u}}u\\ -m\left(z_{G}p-v\right)+Y_{\dot{v}}v & -m\left(z_{G}q+u\right)-X_{\dot{u}}u & m\left(x_{G}p+y_{G}q\right)\\ 0 & -I_{yz}q-I_{xz}p+I_{zz}r+N_{\dot{r}}r & I_{yz}r+I_{xy}p-I_{yy}q-M_{\dot{q}}q\\ I_{yz}q+I_{xz}p-I_{zz}r-N_{\dot{r}}r & 0 & -I_{xz}r-I_{xy}q+I_{xx}p+K_{\dot{p}}p\\ -I_{yz}r-I_{xy}p+I_{yy}q+M_{\dot{q}}q & I_{xz}r+I_{xy}q-I_{xx}p-K_{\dot{p}}p & 0 \end{array}] \end{array},$$

(43) $$D(v)=-diag\{X_{u},Y_{v},Z_{w},K_{p},M_{q},N_{r}\}-diag\{X_{u\left|u\right|}\left|u\right|,Y_{v\left|v\right|}\left|v\right|,Z_{w\left|w\right|}\left|w\right|,K_{p\left|p\right|}\left|p\right|,M_{q\left|q\right|}\left|q\right|,N_{r\left|r\right|}\left|r\right|\},$$

(44) $$G(\eta )=\left[\begin{array}{c} \left(W-B\right)\sin\theta \\ -\left(W-B\right)\cos\theta \sin\phi \\ -\left(W-B\right)\cos\theta \cos\phi \\ -\left(y_{G}W-y_{b}B\right)\cos\theta \cos\phi +(z_{G}W-z_{b}B)\cos\theta \sin\phi \\ (z_{G}W-z_{b}B)\sin\theta +(x_{G}W-x_{b}B)\cos\theta \cos\phi \\ -(x_{G}W-x_{b}B)\cos\theta \sin\phi -\left(y_{G}W-y_{b}B\right)\sin\theta \end{array}\right].$$

All symbols of variables used in the above equations can be explained as follows: m denotes the mass of the UUV, $O_{G}={\left[\begin{array}{ccc} x_{G} & y_{G} & z_{G} \end{array}\right]}^{T}$ is the center of gravity of the UUV, $I_{xx}$, $I_{yy}$, and $I_{zz}$ are the moments of inertia of the UUV about the $Bx_{B}$, $By_{B}$, and $Bz_{B}$ axes, $I_{xy}=I_{yx}$, $I_{xz}=I_{zx}$, and $I_{yz}=I_{zy}$ are the products of inertia, W is the weight of the UUV body expressed in the earth-fixed coordinate, and B is the submerged buoyancy force expressed in the earth-fixed coordinate; *x_b_*, *y_b_*, and *z_b_* are the center of buoyancy of the UUV expressed in the body-fixed coordinate. The partial derivative coefficients ($X_{\dot{u}}$, $Y_{\dot{v}}$, $Z_{\dot{w}}$, $K_{\dot{p}}$, $M_{\dot{q}}$, $N_{\dot{r}}$), the components of linear drag (*X_u_*, *Y_v_*, *Z_w_*, *K_p_*, *N_r_*), and quadratic drag coefficients ($X_{u\left|u\right|}$, $Y_{v\left|v\right|}$, $Z_{w\left|w\right|}$, $K_{p\left|p\right|}$, $M_{q\left|q\right|}$, $N_{r\left|r\right|}$) are the hydrodynamic coefficients which can be directly or indirectly obtained in advance by practical experiments.

Alternatively, the dynamic model equation of the UUV in earth-fixed coordinate E-*XYZ* can also be obtained using the kinematic transformations,

(45) $$\dot{\eta }=J(\eta )v\Leftrightarrow v=J^{-1}(\eta )\dot{\eta },$$

(46) $$\ddot{\eta }=\dot{J}(\eta )v+J(\eta )\dot{v}\Leftrightarrow \dot{v}=J^{-1}(\eta )\left[\ddot{\eta }-\dot{J}(\eta )J^{-1}(\eta )\dot{\eta }\right],$$

to eliminate v and $\dot{v}$ in Equation (40). Hence, the following earth-fixed vector expression of dynamic model can be expressed as

(47) $$M_{\eta }(\eta )\ddot{\eta }+C_{\eta }(\eta ,v)\dot{\eta }+D_{\eta }(\eta ,v)\dot{\eta }+g_{\eta }(\eta )=\tau_{\eta },$$

where

(48) $$\begin{array}{l} M_{\eta }(\eta )=J^{-T}(\eta )MJ^{-1}(\eta )\\ C_{\eta }(v,\eta )=J^{-T}(\eta )\left[C(v)-MJ^{-1}(\eta )\dot{\eta }\right]J^{-1}(\eta )\\ D_{\eta }(v,\eta )=J^{-T}(\eta )D(v)J^{-1}(\eta )\\ g_{\eta }(\eta )=J^{-T}(\eta )g(\eta )\\ \tau_{\eta }(\eta )=J^{-T}(\eta )\tau \end{array}$$

### 5.3. Configuration of Thrusters

**Figure 12:**
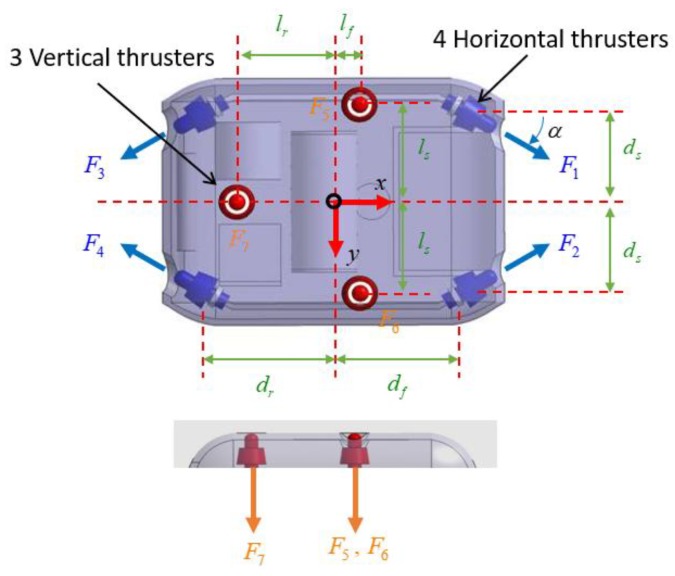
Layout of thrusters of the UUV.

In this paper, the UUV was equipped with seven thrusters as shown in Figure 12. The UUV did not have rudders; thus, its motion was only affected by the thrusters. Four horizontal thrusters *T*_1_, *T*_2_, *T*_3_, and *T*_4_, which were installed at the bow and the stern part with inclined angle α, were responsible for the motions along the horizontal plane. Meanwhile, three vertical thrusters *T*_5_, *T*_6_, and *T*_7_ were responsible for the motions along the vertical plane.

Since the UUV was equipped with seven thrusters and controlled in 6-DOF, the relationship between the required force in each DOF and the forces was

(49) $$\tau_{th}=T\tau_{u},$$

in which $\tau_{th}$ is the desired force in the different DOF, T is the thruster configuration matrix, and $\tau_{u}$ is the desired force of each thruster. $\tau_{th}$ and $\tau_{u}$ are defined as

(50) $$\tau_{th}={\left[\begin{array}{cccccc} F_{Tx} & F_{Ty} & F_{Tz} & M_{Tx} & M_{Ty} & M_{Tz} \end{array}\right]}^{T},$$

(51) $$\tau_{u}={\left[\begin{array}{ccccccc} F_{1} & F_{2} & F_{3} & F_{4} & F_{5} & F_{6} & F_{7} \end{array}\right]}^{T}.$$

The thruster configuration matrix T can be expressed as follows:

(52) $$T=\left[\begin{array}{ccccccc} c\;\alpha & c\;\alpha & -c\;\alpha & -c\;\alpha & 0 & 0 & 0\\ s\;\alpha & -s\;\alpha & s\;\alpha & -s\;\alpha & 0 & 0 & 0\\ 0 & 0 & 0 & 0 & 1 & 1 & 1\\ 0 & 0 & 0 & 0 & -l_{s} & l_{s} & 0\\ 0 & 0 & 0 & 0 & -l_{f} & l_{f} & l_{r}\\ \begin{array}{l} d_{s}\;c\alpha \\ \;\;\;+d_{f}\;s\alpha \end{array} & \begin{array}{l} -d_{s}\;c\alpha \\ \;\;\;-d_{f}\;s\alpha \end{array} & \begin{array}{l} -d_{s}\;c\alpha \\ \;\;\;-d_{r}\;s\alpha \end{array} & \begin{array}{l} d_{s}\;c\alpha \\ \;\;\;+d_{r}\;s\alpha \end{array} & 0 & 0 & 0 \end{array}\right],$$

where *l_s_*, *l_f_*, *l_r_*, *d_s_*, *d_f_*, *d_r_* are the lengths of the arm that create momentum in roll, pitch, and yaw, and $\alpha =30^{0}$ is the angle thruster which located in the *xy*-plane.

## 6. Simulation Results and Discussion

### 6.1. Simulation Procedure

**Figure 13:**
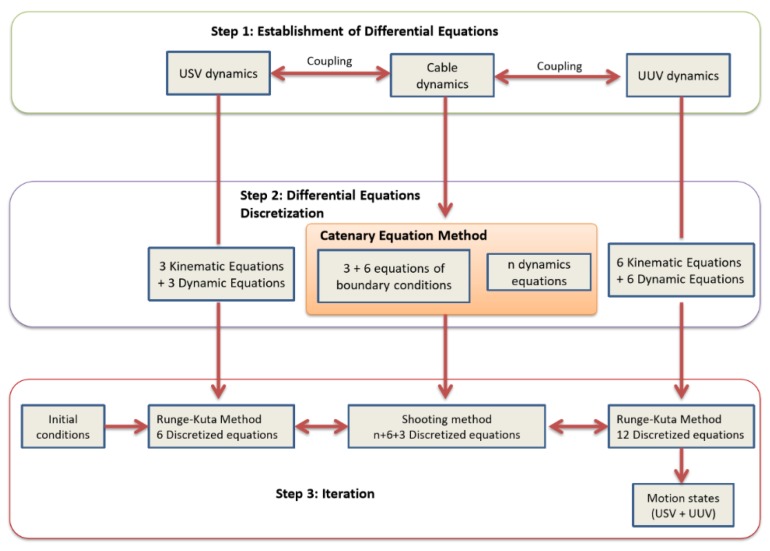
The model-based simulation process.

As the nonlinear dynamic equations are complex and difficult to solve analytically, the numerical simulation approach was adopted to simulate the motion of the USV–UC–UUV coupling system. For this, a model-based simulation process is proposed as shown in Figure 13. After the establishment of the respective dynamic equations of the coupled USV–UC–UUV system, the dynamic equations were further discretized for numerical simulation. Thus, several iteration methods were applied to solve the discretized equations.

In order to solve the nonlinear differential equations, different iteration methods were applied to the respective USV, UUV, and UC dynamic equations due to their different dynamic characteristics. Usually, the Runge–Kutta method is well known to solve the differential equations of USV and UUV dynamics, while the shooting method is used to solve the partial differential equations of UC dynamics. For the dynamic behavior of the UC, the partial differential Equation (26) under the boundary conditions Equations (31) and (32) could be solved numerically using the shooting method. For this, the UC was divided into *n* segments (or nodes) equally, such that the shooting method could be used by discretizing the UC dynamics using both the time segment (δt) and the cable length segment (δs). Note that the length of the connecting UC was fixed when the USV and UUV moved forward in the water. In this case, the length of each segment of the UC was fixed in the finite difference method.

The finite difference in Equation (26) involves *n* cable nodes, and, according to the boundary conditions in Equations (31) and (32), there are 3*n* + (6 + 3) cable node variables in total. Because the UUV is a rigid body, 12 motion states were used to describe its dynamics. The USV had 6 motion states to describe the dynamics. Thus, for the combined USV–UC–UUV system, there were in total 6 + (3*n* + 6 + 3) + 12 dynamic equations to solve the 6 + (3*n* + 6 + 3) + 12 motion states, as described below.

(53) $$\begin{array}{l} U_{whole}=\{u_{1:n},\left(X_{USV},Y_{USV},\psi_{USV},u_{USV},v_{USV},r_{USV}\right),\;\\ \;\;\;\;\;\;\;\;\;\;\;\;\;\;\;\;\;{\left(X_{UUV},Y_{UUV},Z_{UUV},\phi_{UUV},\theta_{UUV},\psi_{UUV},u_{UUV},v_{UUV},w_{UUV},p_{UUV},q_{UUV},r_{UUV}\right)\}}^{T} \end{array}$$

**Figure 14:**
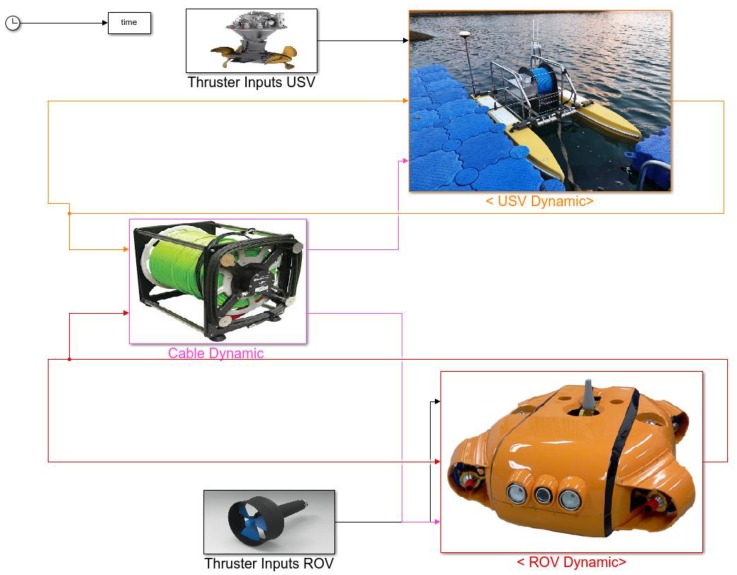
Block diagram of the simulation program.

To calculate the motion states in Equation (53), the initial condition of the system ($U_{whole}^{0}$) should be provided for further calculation. Then, the previous iteration result ($U_{whole}^{k}$) can be used as the initial guess for the next iteration ($U_{whole}^{k+1}$). In this paper, an integrated simulation model was made to represent the dynamics of the complete USV–UC–UUV system in the time domain. The simulation model in Matlab-Simulink consisted of the USV dynamics, the UC dynamics, and the UUV model as shown in Figure 14.

The parameters for simulation of the complete USV–UC–UUV are shown in Table 1. The detailed parameters of the USV and UUV used in this study were given in References [20,21,22], respectively.

It is challenging to simulate the complete USV–UC–UUV system as it is a poorly damped system and the dynamics are highly nonlinear. Based on the simulation scheme introduced in Figure 13, the model-based motion simulations are further implemented in the later sections. The three following simulations were performed:

- **Simulation 1**: The dynamic behavior of the complete USV (fixed position)–UC–UUV (turning motion);
- **Simulation 2**: The dynamic behavior of the complete USV (turning motion)–UC–UUV (fixed position);
- **Simulation 3**: The dynamic behavior of the complete USV (forward motion)–UC–UUV (sideward motion).

### 6.2. Simulation 1

**Figure 15:**
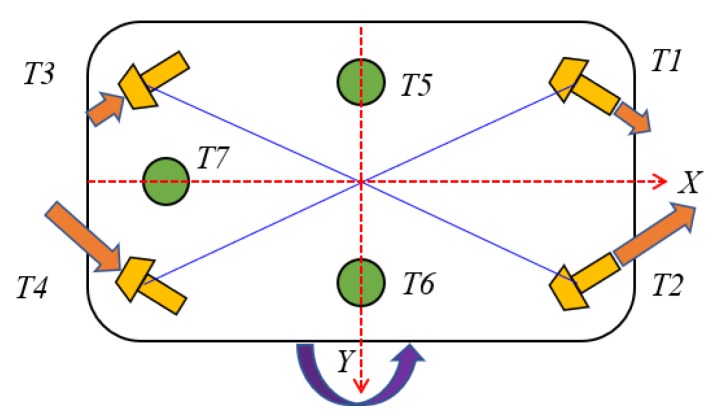
Thruster directions of the UUV in the turning motion.

In the first simulation, the motion of the coupled UUV and UC system in the horizontal plane was studied. The USV was assumed to stay at a position by using a perfect dynamic positioning controller such that the USV-induced motion was ignored in this case. At this time, the UUV performed a turning motion as shown in Figure 15. In this simulation, the simulation duration was 35 s, with a sampling time of 0.01 s. The initial position of the UUV in the earth-fixed coordinate was $\eta_{UUV}=\left(70,0,50,0,0,0\right)$, while the initial velocity of the UUV expressed in body-fixed coordinate was $\nu_{UUV}=\left(0.2,0,0,0,0,0\right)$. On the contrary, the USV was assumed at the stable position (0, 0, 0) by using a strong controller.

A shown in Figure 15, to force the UUV into a clockwise turning motion, the four horizontal thrusters T1, T2, T3, and T4 were applied using different values such as T1 = 11.5 N, T2 = 12 N, T3 = −11.5 N, and T4 = −12 N. Meanwhile, the three thrusters T5, T6, T7 were set to 0 N to achieve a pure turning motion in this simulation.

**Figure 16:**
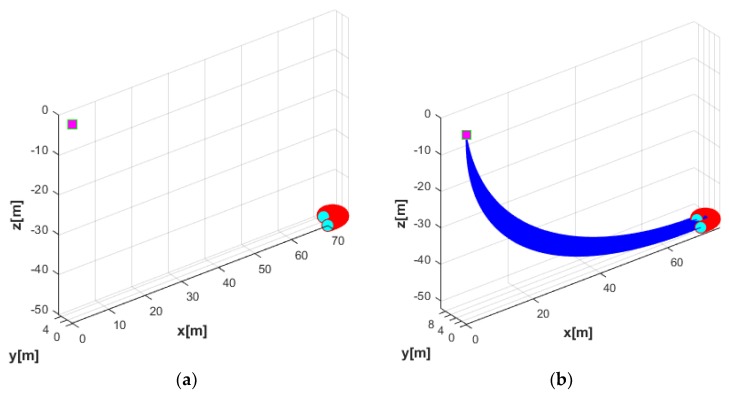
Trajectories of the complete systems: (**a**) trajectory of UUV in the turning motion without the UC; (**b**) trajectory of UUV in the turning motion with the UC.

In general, the UUV drag increases as the length of the UC increases. Thus, the maximum affordable UC length for the UUV needs to be designed according to the power capacity of the UUV. The trajectories of the UUV runs in terms of the turning motion without the UC and with the UC are shown in Figure 16.

**Figure 17:**
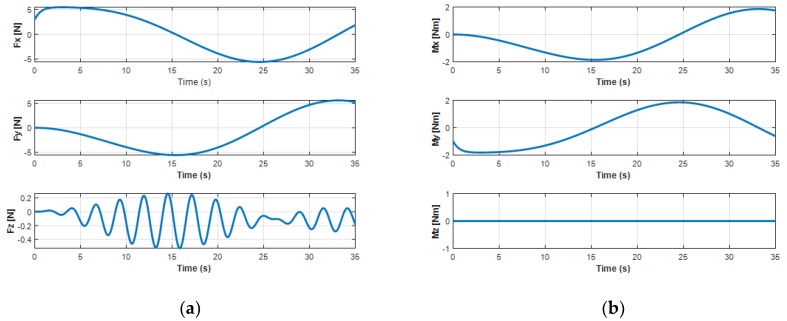
Cable forces and moments affecting UUV turning motion: (**a**) forces of the UC on the UUV; (**b**) moments of the UC on the UUV.

The variation of the force and moment of the UC acting upon the turning motion of the UUV is shown in Figure 17. It shows that the oscillatory heave force *F_cz_* and the roll moment *M_cx_* from the UC caused the oscillators to achieve the heave velocity and the roll motion of the UUV. In addition, the surge force *F_xz_* initially increased and then decreased due to the negative value right after the UUV turning to the left side, while the sway force *F_cy_* decreased at the beginning and then increased to a positive value when the UUV underwent the turning motion.

**Figure 18:**
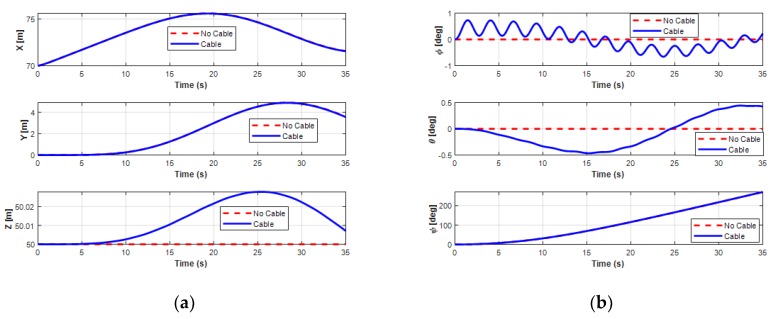
Simulation results of the position and orientation behaviors of the UUV turning: (**a**) position of the UUV; (**b**) orientation of the UUV.

The effects of the UC on the position and orientation of the UUV are clearly shown in Figure 18. Obviously, when operating the UUV and the UC coupling system in the turning motion, the state variables of the UUV were significantly affected.

**Figure 19:**
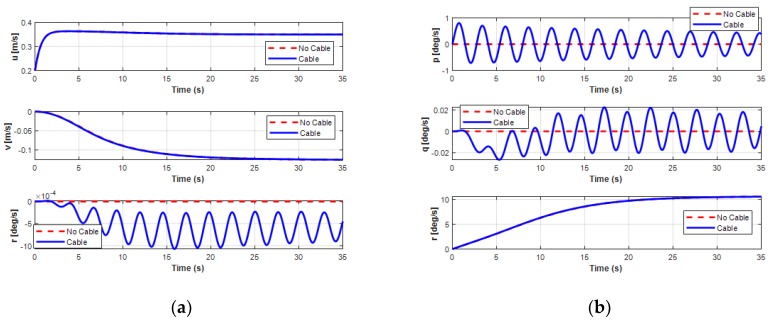
Simulation results of the linear and angular velocities behaviors of the UUV turning: (**a**) linear velocities of the UUV; (**b**) angular velocities of the UUV.

For the velocities of the UUV, the UUV initially moved straight for about 10 s, then turned leftward, and finally moved backward. In this motion, the surge velocity increased from the initial value 0.2 m/s and then decreased to a constant speed 0.37 m/s, while the sway speed of the UUV slightly decreased from 0 m/s to a negative small steady value of about −0.12 m/s, as shown in Figure 19. For the heave velocity and roll motion, oscillations are shown. Moreover, the pitch motion is also affected because of the UC with a negative decrease negatively initially before increasing to a positive angle. However, the results also show that the interaction of the UC and the UUV did not affect the surge motion, sway motion, and yaw motion of the UUV.

### 6.3. Simulation 2

**Figure 20:**
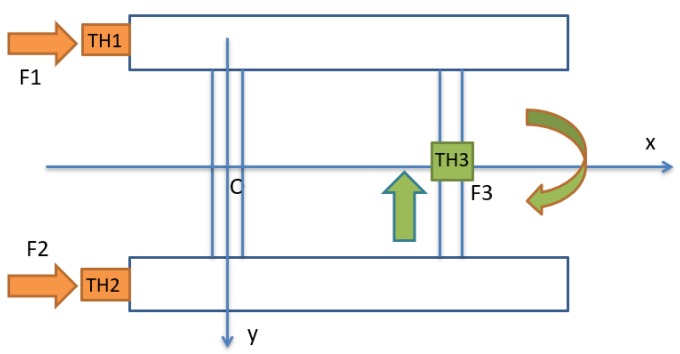
Thruster directions of the USV in turning motion.

In the second simulation, the motion of the coupled USV and UC system in the horizontal plane was analyzed with time varied. Unlike the first simulation, the 3-DOF USV firstly moved straight in the forward direction for about 15 s from the origin of the earth-fixed coordinate $\eta_{USV}=\left(0,0,0,0,0,0\right)$, then turned left afterward, while the UUV was assumed to maintain position (50, 0, 70). Furthermore, the initial velocity of the USV was $\nu_{USV}=\left(0.1,0,0,0,0,0\right)$ and the simulation duration was 25 s, with a sampling time of 0.01 s in this case. Figure 20 shows the thruster directions of the USV during its turning motion. In order to simulate the turning motion of the USV, two stern thrusters TH1 and TH2 were applied with different values of TH1 = 10 N and TH2 = 9 N, while the bow thruster TH3 was set to 0 N.

**Figure 21:**
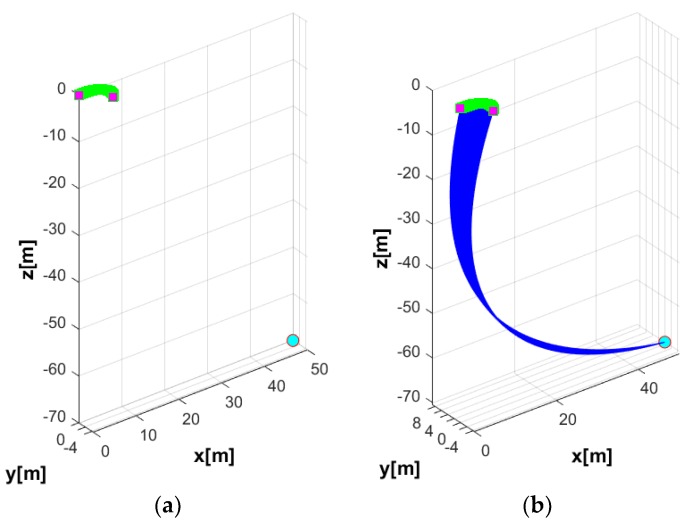
Trajectory of the complete systems: (**a**) trajectory of USV in the turning motion without the UC; (**b**) trajectory of USV in the turning motion with the UC.

**Figure 22:**
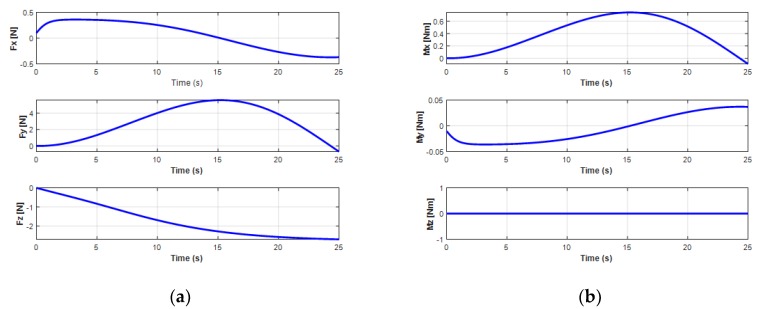
Cable forces and moments affecting USV turning motion: (**a**) forces of the UC on the USV; (**b**) moments of the UC on the USV.

**Figure 23:**
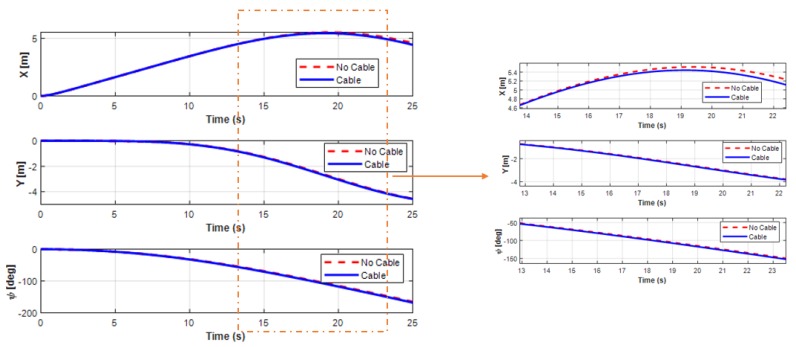
Simulation results of the position behaviors in the USV turning motion.

**Figure 24:**
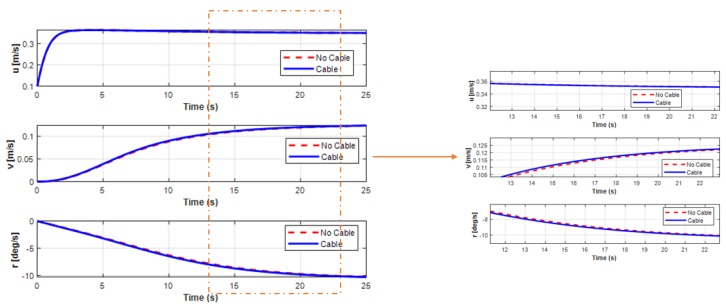
Simulation results of the velocity behaviors in the USV turning motion.

Figure 21 shows the trajectory of the USV during the turning motion, either with or without the UC. In this study, the proposed system consisted of the USV connected to the UUV using the UC. Thus, the forces generated by the motion of the UC could affect the motion of the combined vehicles. The forces and moments of the UC affecting the USV during the turning motion are presented in Figure 22. It shows that the exerting force of the UC on the USV seemed significant when the UUV underwent the turning motion. However, all the state variables of USV (surge, sway, and yaw motions) were not affected much, as shown in Figure 23 and Figure 24. The corresponding phenomena can be explained by the size of the USV being relatively large compared to that of the UUV, while the movement of the UC and the UUV had a small effect on the behavior of the USV.

### 6.4. Simulation 3

In this simulation, the dynamic behaviors of the complete USV–UC–UUV system were analyzed to show the effects of the UC on USV and UUV motions. The USV went straight in the forward direction, while the UUV undertook a sideward motion. Similarly to simulation 1, a time interval of 0.01 s and a simulation time of 35 s were set. The initial positions of the connecting point to the UUV (position of the UUV in the earth-fixed coordinate) were set at (70 m, 0 m, 50 m) while the end point at the free surface near the USV was assumed set at (0 m, 0 m, 0 m). The initial velocities of both USV and UUV in the body-fixed coordinate were $\nu_{USV}=\left(0.1,0,0,0,0,0\right)$ and $\nu_{UUV}=\left(0.2,0,0,0,0,0\right)$, respectively.

**Figure 25:**
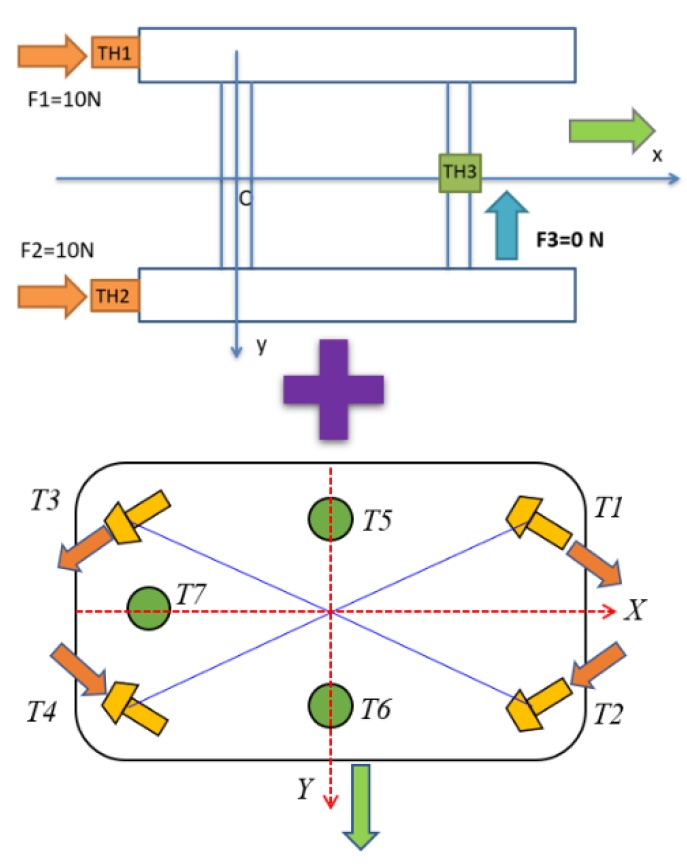
Thruster directions of the USV and the UUV.

Figure 25 shows the thruster directions of the USV and the UUV during their motions. In order to simulate the forward motion of the USV, the thrust forces of the two stern thrusters TH1 and TH2 were set to 10 N, while the bow thruster TH3 was set to 0 N. For the case of sideward motion of the UUV, the thrust forces of T1 and T3 were set to 10 N, while the thrusters T2 and T4 were set to −10 N, as shown in Figure 25. Meanwhile, thrusters T5, T6, and T7 were set to 0 N to have sideward motion only.

**Figure 26:**
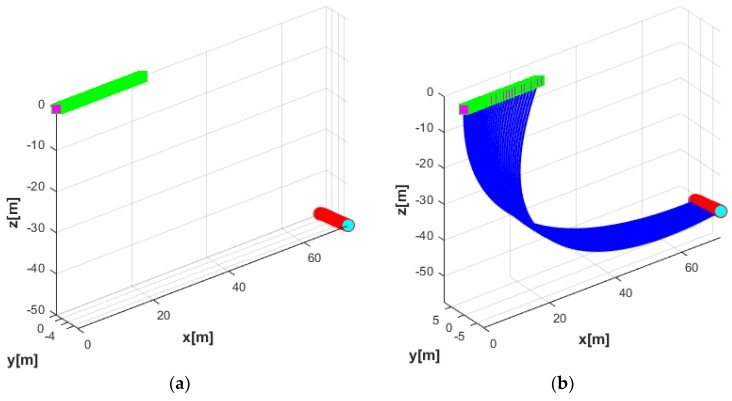
Trajectory of the complete systems: (**a**) trajectory of the USV and the UUV without the UC; (**b**) trajectory of the USV and the UUV with the UC.

**Figure 27:**
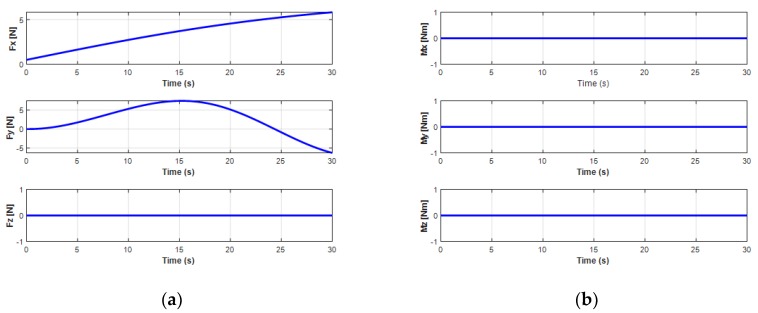
Cable forces and moments effecting to the USV doing the forward motion: (**a**) forces of the UC on the USV; (**b**) moments of the UC on the USV.

**Figure 28:**
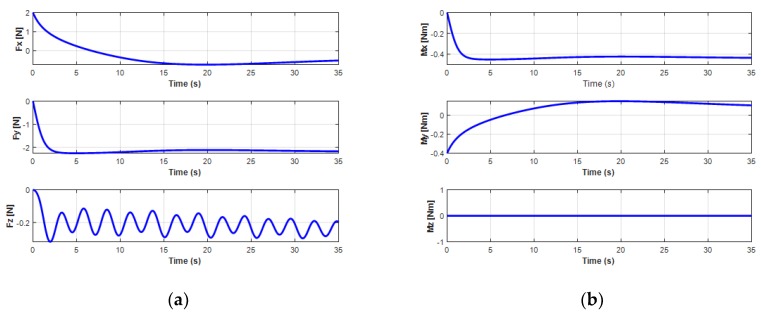
Cable forces and moments effecting to the UUV doing the sideward motion: (**a**) forces of the UC on the UUV; (**b**) moments of the UC on the UUV.

For the above case, Figure 26 shows the trajectories of the USV and UUV without the UC and with the UC interacting force. According to the simulation results, in order to maintain the depth of the UUV during operation of the complete USV–UC–UUV system, the speed difference between the USV and the UUV should be confined. The UC tension causes additional drag on the USV and UUV motions, which affect the behavior of the vehicles. The variations of the UC force and moment at the tow points (upper and lower points) are presented for the USV and UUV, as shown in Figure 27 and Figure 28, respectively. Comparing Figure 27 with Figure 28, it is shown that the force of the UC on the UUV is more significant than on the USV.

**Figure 29:**
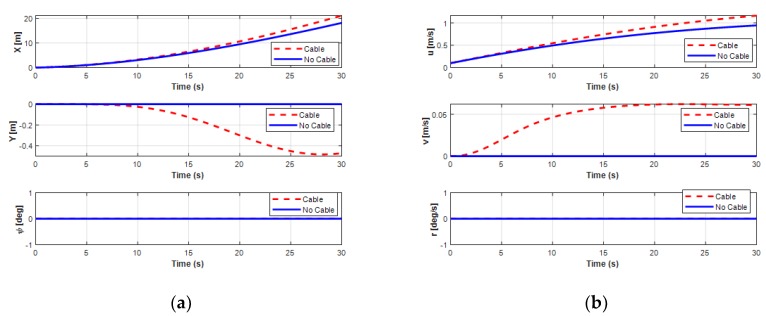
Simulation results of position and velocity of the USV doing the forward motion: (**a**) position of the USV; (**b**) velocity of the USV.

**Figure 30:**
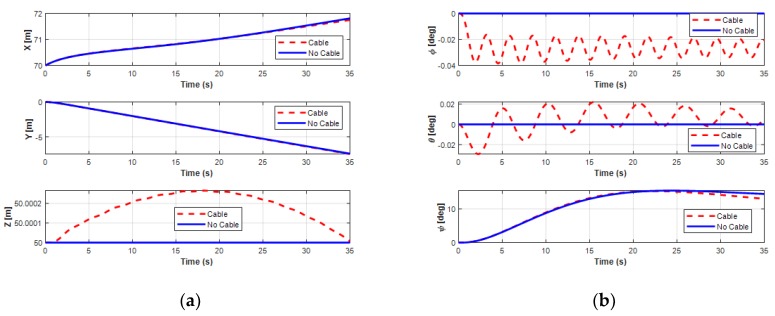
Simulation results of position and orientation of the UUV doing the sideward motion: (**a**) position of the UUV; (**b**) orientation of the UUV.

**Figure 31:**
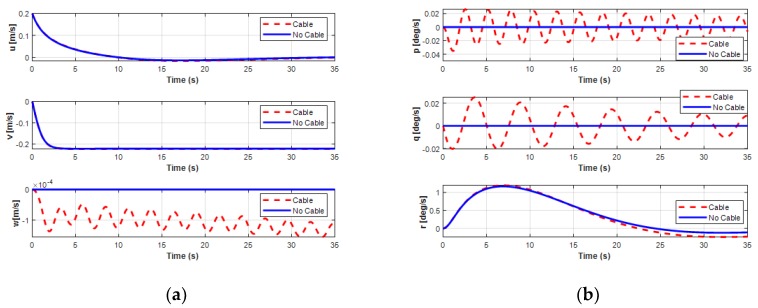
Simulation results of linear and angular velocities of the UUV doing the sideward motion: (**a**) linear velocities of the UUV; (**b**) angular velocities of the UUV.

Unlike simulations 1 and 2, by considering the different motion operations on the horizontal plane of the USV and the UUV, the analysis on the dynamic behavior of both the USV and the UUV affected by the interaction of the UC is presented in this simulation. The effects of the UC on the USV and the UUV are presented in Figure 29, Figure 30 and Figure 31. For USV motion, the USV was set to move in a straight line with the velocity *V_usv_* = 0.25 m/s; after 25 s, the velocity increased to 0.8 m/s as shown in Figure 29. The results also show that the USV moved faster with the connected UC because the pushed forces of the surge force, *F_x_*, increased gradually.

For the sideward motion of the UUV, the UUV moved leftward for 7.5 m in 35 s with a sway velocity of about 0.23 m/s, as shown in Figure 30 and Figure 31. The results in these figures show that the UUV shifted a little in surge motion while moving sideward due to the initial surge velocity of the UUV set to 0.2 m/s. For this motion, the depth of the UUV slightly decreased. Figure 30 shows the significant effect of the UC on the depth, roll, and pitch motion modes. In particular, both the pitch and heave motions of the UUV were significantly oscillatory because of the heave force *F_cz_*. The UUV moved in the sway direction with up and down oscillations and changed the pitch angle of the UUV during simulation time. This occurred because the heave velocity w and the pitch angle *θ* of the UUV regularly oscillated. Furthermore, the results of the surge, sway, and yaw motions of the UUV showed similar motion compared to previous ones without UC.

In summary, the simulations results show that the effects of the connected UC to the vehicles were big, especially for the UUV motions. The simulations could be very helpful when designing the capacity of the thrusters for the UUV and USV.

## 7. Conclusions

In this paper, a new mathematical modeling of a coupling system of a USV connected to a UUV using a UC was presented. To do so, analysis of the dynamics was firstly performed on each system, and the total coupled system dynamics was then studied. Because the UC connected the USV and the UUV, the dynamic equation of the UC was derived using the catenary equation. To analyze the behavior of the UC, the shooting method was applied. To demonstrate the application of the proposed equations, model-based motion simulations of the coupling system were performed. Computer simulations were conducted to analyze the interacting forces of the UC with the USV and UUV systems. In the simulation, the maneuvering behavior of the USV with the UC, the maneuvering behavior of the UUV with the UC, and the maneuvering behavior of the coupled USV–UC–UUV system were investigated and their results were discussed. Moreover, the variation of the UC forces and moments at the tow points and the configuration of the UC were analyzed.

The simulation results revealed that the UC significantly affected the motion of both the USV and the UUV in all cases (especially the UUV). The results also showed that the UC tension caused additional drag forces on the USV and UUV, and they affected the motion of both vehicles, while the variation of the configuration of the UC could result in the vehicle getting tangled, especially when the coupled system moves in currents. Based on suitable assumptions, the numerical model developed in the paper could numerically simulate the motions of the vehicles with the UC in the ocean environment. It is believed that the simulation results may provide useful guidance and reference for real USV–UC–UUV systems in design and operation. Using the results of the analysis on the interaction forces between the systems, it would be helpful to design the capacity of actuators for the USV or UUV.

Future work can be extended by taking into account the motion of the USV–UC–UUV system under the effect of various speeds of underwater currents, with verification via water tests. Furthermore, an analysis on the controller will be designed to reduce the effects of the UC.

## Figures and Tables

**Table 1:** The parameters for the simulation.

Properties	Units	Symbols	Values
USV parameters			
Length of USV	m	$L_{USV}$	2.5
Breadth of USV	m	$B_{USV}$	0.63
Draft of USV	m	$D_{USV}$	0.201
Mass of USV	kg	$M_{USV}$	220.4
Center of gravity	m	$X_{G_{USV}}$	−0.076
Diameter of propeller	m	$D_{P_{USV}}$	0.122
**Cable parameters**			
Length of cable	m	*L_c_*	100
Cable density	kg/m^3^	*ρ_c_*	662.2
Diameter of cable	m	*d_c_*	0.025
Axial stiffness	N	*EA*	3 × 10^4^
Guess for end force (we know this guess is wrong)	N	*F_end_*	(4, 5, 100)
Weight per length of cable	kg/m	*w_c_*	0.5
Mesh frame of cable	m	*s*	0:0.1:100
Normal drag coefficient	-	*C_n_*	1.2
Tangential drag coefficient	-	*C_f_*	0.062
Tension rigidity	N	*T*	Inextensible
**Environment parameters**			
Sea state	-	-	Calm sea
Water current velocity	m/s	*v_w_*	0.1
Seawater density	kg/m^3^	*ρ_w_*	1000
**UUV parameters**			
Dimension of UUV	mm	$L_{UUV}\times B_{UUV}\times H_{UUV}$	560 × 750 × 280
Weight of UUV	kg	$m_{UUV}$	80
Center of gravity	m	$X_{G_{UUV}}$	(0, 0, −0.06)
Center of buoyancy	m	$X_{B_{UUV}}$	(0, 0, 0)
Mass moment of inertia *x*-axis	kg.m^2^	$I_{xx}$	6.9
Mass moment of inertia *y*-axis	kg.m^2^	$I_{yy}$	26.1
Mass moment of inertia *z*-axis	kg.m^2^	$I_{zz}$	23.2
